# Genome-wide DNA methylation profiling integrated with gene expression profiling identifies *PAX9* as a novel prognostic marker in chronic lymphocytic leukemia

**DOI:** 10.1186/s13148-017-0356-0

**Published:** 2017-05-30

**Authors:** Lata Rani, Nitin Mathur, Ritu Gupta, Ajay Gogia, Gurvinder Kaur, Jaspreet Kaur Dhanjal, Durai Sundar, Lalit Kumar, Atul Sharma

**Affiliations:** 1 Laboratory Oncology Unit, Dr. B.R.A.IRCH, All India Institute of Medical Sciences (AIIMS), Ansari Nagar, New Delhi, 110029 India; 2 Department of Medical Oncology, Dr. B.R.A.IRCH, All India Institute of Medical Sciences (AIIMS), Ansari Nagar, New Delhi, 110029 India; 3 Department of Biochemical Engineering and Biotechnology, DBT-AIST International Laboratory of Advanced Biomedicine (DAILAB), Indian Institute of Technology (IIT) Delhi, Hauz Khas, New Delhi, 110016 India

**Keywords:** Promoter methylation, *PAX9*, Circadian rhythm, Transcription factors

## Abstract

**Background:**

In chronic lymphocytic leukemia (CLL), epigenomic and genomic studies have expanded the existing knowledge about the disease biology and led to the identification of potential biomarkers relevant for implementation of personalized medicine. In this study, an attempt has been made to examine and integrate the global DNA methylation changes with gene expression profile and their impact on clinical outcome in early stage CLL patients.

**Results:**

The integration of DNA methylation profile (*n* = 14) with the gene expression profile (*n* = 21) revealed 142 genes as hypermethylated-downregulated and; 62 genes as hypomethylated-upregulated in early stage CLL patients compared to CD19+ B-cells from healthy individuals. The mRNA expression levels of 17 genes identified to be differentially methylated and/or differentially expressed was further examined in early stage CLL patients (*n* = 93) by quantitative real time PCR (RQ-PCR). Significant differences were observed in the mRNA expression of *MEIS1*, *PMEPA1*, *SOX7*, *SPRY1*, *CDK6*, *TBX2*, and *SPRY2* genes in CLL cells as compared to B-cells from healthy individuals. The analysis in the *IGHV* mutation based categories (Unmutated = 39, Mutated = 54) revealed significantly higher mRNA expression of *CRY1* and *PAX9* genes in the *IGHV* unmutated subgroup (*p* < 0.001). The relative risk of treatment initiation was significantly higher among patients with high expression of *CRY1* (RR = 1.91, *p* = 0.005) or *PAX9* (RR = 1.87, *p* = 0.001). High expression of *CRY1* (HR: 3.53, *p* < 0.001) or *PAX9* (HR: 3.14, *p* < 0.001) gene was significantly associated with shorter time to first treatment. The high expression of *PAX9* gene (HR: 3.29, 95% CI 1.172–9.272, *p* = 0.016) was also predictive of shorter overall survival in CLL.

**Conclusions:**

The DNA methylation changes associated with mRNA expression of *CRY1* and *PAX9* genes allow risk stratification of early stage CLL patients. This comprehensive analysis supports the concept that the epigenetic changes along with the altered expression of genes have the potential to predict clinical outcome in early stage CLL patients.

**Electronic supplementary material:**

The online version of this article (doi:10.1186/s13148-017-0356-0) contains supplementary material, which is available to authorized users.

## Background

Chronic lymphocytic leukemia (CLL) arises from a malignant clone of B cells due to altered control of apoptosis and dysregulated rate of proliferation. Its progression is characterized by clonal proliferation and accumulation of mature neoplastic CD5^+^ B lymphocytes [1]. The clinical course of CLL patients is extremely variable with some patients progressing rapidly as compared to others and ultimately, requiring therapeutic intervention. Several biomarkers including immunoglobulin heavy chain variable (*IGHV*) gene mutations that segregate CLL patients into low and high-risk clinical groups are widely used to assess the prognosis of these patients. Low-risk patients generally display mutated *IGHV* gene, low CD38, and low ζ chain associated protein kinase-70 (*ZAP-70*) expression, while high-risk cases exhibit the reverse pattern [2–6].

Altered DNA methylation is one of the hallmark events in cancer. The first evidence of DNA methylation in CLL was presented by Wahlfors et al. [7] in which a global loss of methylation was reported. In addition to global hypomethylation, hypermethylation of individual gene promoters has also been reported in CLL [7–11]. Methylation of *TWIST2* and *ZAP-70* exhibited a strong association with the *IGHV-*mutated status [9, 12] whereas methylation of *HOXA4* gene was predominantly associated with the *IGHV* unmutated status [13]. Further studies employing genome wide methylation profiling technologies have revealed association of differential methylation patterns with prognostic subgroups based on the *IGHV* mutation status [14–16], CD38 levels [17], *ZAP-70* levels [16], immunogenetic subsets [18], and 17p-deletion status [19].

Earlier, DNA hypermethylation was thought to affect the expression of a gene negatively but the emerging research has suggested that the function and effect of DNA methylation is contextual, and the relationship between DNA methylation and transcription is more complex [20]. In CLL, although association of differential methylation patterns with specific prognostic subgroups in earlier reports highlights the potential of altered gene methylation as a tool to predict clinical outcome, further research is required to establish the relationship between the epigenome and the transcriptome. The present study was carried out to correlate the DNA methylation patterns with gene expression profile and to assess the prognostic implications of such correlations on clinical outcome in 93 early stage CLL patients.

## Methods

### Patient selection

Treatment naive early stage (Rai 0-II) CLL patients (*n* = 100) were enrolled in the study after obtaining informed consent as per the guidelines of the institute ethics committee. According to the staging criteria outlined by Rai et al. [21], 24 patients were in stage 0, 33 were in stage I and 43 were in stage II. Fourteen randomly selected CLL samples and pooled CD19+ B-cells from 10 healthy individuals were profiled for methylation. Gene expression profiling was carried out in 21 CLL samples and pooled CD19+ B-cells from 10 healthy individuals. All the CLL samples had at least ≥65% CLL phenotype cells. The clinical and laboratory characteristics of the CLL patients analysed using methylation and gene expression arrays are provided in Table 1. The mRNA expression of 17 of the genes identified to be differentially methylated and /or differentially expressed was validated using SYBR-green based RQ-PCR in 93 (Unmutated = 39, Mutated = 54) CLL patients. The median age of the CLL patients was 60 years (range 35–80 years). With a median follow-up time of 22 months (range 1-124 months), 46 patients required treatment [median time to treatment: 14 months (range 0–92 months)] and 18 patients died. On the basis of international prognostic index (IPI) score [22], 11/93 patients were assigned as low risk, 34/93 as intermediate risk, 43/93 as high risk, and 5/93 as very high risk patients.

**Table 1:** Clinical and laboratory characteristics of the CLL patients evaluated using methylation and gene expression arrays

Characteristics of the patients										
Sr. No	Sample ID	Methylation array	GE array	Rai stage	Age	Tumor Percentage	IGHV status	β2M(mg/L)	17p Deletion	IPI Score
1	S1	√		0	41	67.2	UM	6.56	Absent	4
2	S2	√	√	0	63	69	UM	4.06	Absent	4
3	S3	√	√	0	69	88.6	UM	8.42	Absent	5
4	S4	√	√	I	45	95.4	UM	4.6	Present	9
5	S5	√	√	I	60	93.5	UM	3.37	Absent	3
6	S6	√	√	II	50	66	UM	4.78	Absent	5
7	S7	√		II	59	97.2	M	3.54	Absent	3
8	S8	√		0	57	95.3	M	6.49	Absent	2
9	S9	√		II	65	92.8	M	4.58	Absent	3
10	S10	√	√	0	61	69.3	M	3.3	Absent	0
11	S11	√	√	II	65	97.2	M	4.48	Present	7
12	S12	√	√	II	67	98	UM	5.69	Absent	6
13	S13	√	√	II	48	92	UM	4.42	Absent	5
14	S14	√		I	58	95.62	UM	6.31	Absent	5
15	S15		√	I	46	79.4	UM	6.78	Absent	5
16	S16		√	II	59	73	UM	4.43	Absent	5
17	S17		√	I	40	90	UM	7.74	Absent	5
18	S18		√	I	67	79.3	UM	2.59	Absent	4
19	S19		√	I	51	80	M	5.44	Absent	3
20	S20		√	0	57	72.5	M	2.91	Absent	0
21	S21		√	II	65	68.6	M	7.1	Absent	2
22	S22		√	II	52	66	M	3.2	Absent	1
23	S23		√	I	60	92	M	6.56	Absent	3
24	S24		√	I	44	85.1	M	3.62	Absent	3
25	S25		√	II	68	93	M	6.47	Absent	4
26	S26		√	II	80	68.4	M	6.46	Absent	4

Abbreviations used: *GE* Gene expression, *UM* Unmutated, *M* Mutated , *β 2 M* Beta 2 Microglobulin, *IPI* International Prognostic index

### IGHV mutation status

*IGHV* gene family usage was evaluated as per BIOMED-2 protocol [23] and the patients were assigned to *IGHV* mutated or unmutated subgroups based on the *IGHV* sequence homology (cut-off = 98%) as determined by the international ImmunoGeneTics database (IMGT; http:// imgt.cines.fr, Montpellier, France).

### Methylated CpG island microarrays

Genomic DNA was extracted from the peripheral blood mononuclear cells (PBMC) of CLL patients (*n* = 14) and CD19^+^ sorted cells pooled from 10 healthy individuals. To isolate the CD19+ cells, mononuclear cells isolated from peripheral blood of healthy individuals were incubated with CD19 + magnetic microbeads and processed according to the manufacturer’s protocol (Milteneyi Biotech, Gladbach, Germany). In healthy individuals, CD19+ cells constitute 2-3% of the leukocyte fraction and therefore, sorted CD19+ B-cells from healthy individuals were used. In the CLL samples evaluated for microarrays, CD19+ cells constituted at least ≥65% of the leukocytes and the PBMC fraction from CLL patients was used for the study.

For methylated CpG island microarrays, 6 μg of genomic DNA was digested with *Mse* I restriction enzyme (New England Biolabs Inc., Ipswich, MA, USA) and labelled with anti-5 methyl cytidine antibody (Abcam, Cambridge, UK). One fraction of the labelled DNA was immunoprecipitated while the other was used as input DNA. Both the input and immunoprecipitated fractions were purified followed by whole genome amplification (WGA, Sigma Aldrich, St. Louis, MO, USA), labelled with Cy3- and Cy5-dUTP, respectively, and hybridized on 1x244K human promoter chIP-on-chip microarray slides as per the manufacturer’s recommendations (Agilent Technologies, Santa Clara, CA, USA). The slides were washed and scanned on the Agilent DNA microarray scanner D and the data was extracted with Feature Extraction® software FE version 11.5 (Agilent Technologies, Santa Clara, CA, USA).

### Gene expression microarray

Total RNA obtained from PBMC of CLL patients (*n* = 21) and CD19+ sorted cells pooled from 10 healthy individuals was amplified and simultaneously labelled with Cy3-CTP using low input quick amp labelling kit (Agilent Technologies, Santa Clara, CA, USA). The labelled product was finally hybridized to SurePrint G3 Human Gene Expression 8x60K microarray slide as per manufacturer’s recommendation (Agilent Technologies, Santa Clara, CA, USA). The slides were washed and scanned on the Agilent DNA microarray scanner D and the data was extracted with Feature Extraction® software FE version 11.5 (Agilent Technologies, Santa Clara, CA, USA). These samples included seven CLL samples profiled for DNA methylation status.

### Bisulfite genome sequencing

**Fig. 1:**
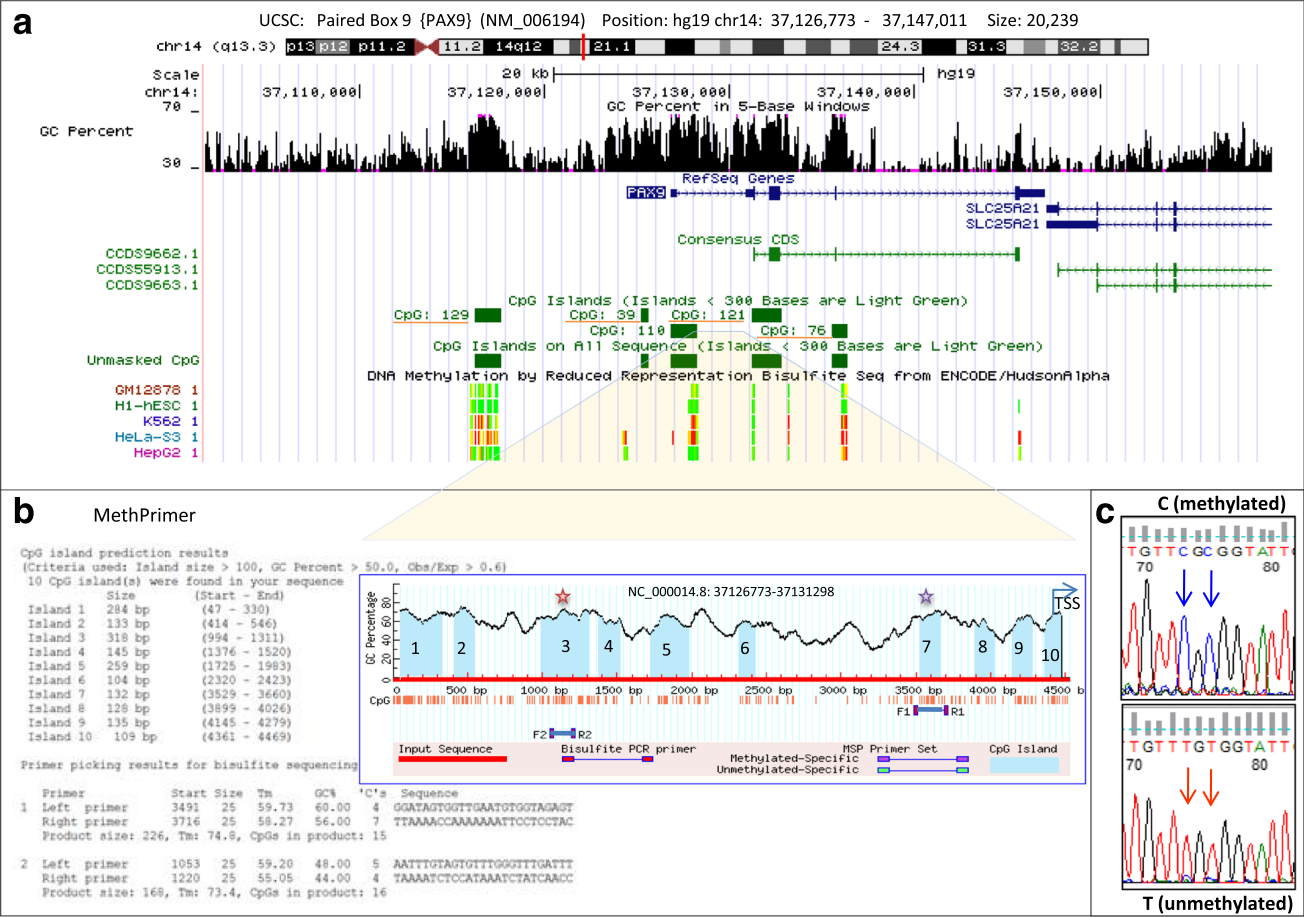
Location of CpG islands studied for *PAX9* gene methylation. **a** UCSC browser view of *PAX9* gene (chromosome 14q13.3). The probes used for methylation microarrays were specific for CpG islands 121, 129, 39, and 76. **b** MethPrimer based CpG prediction and primer design for bisulfite gene sequencing for two CpG islands (3 and 7) located at *PAX9* upstream region. **c** Bisulfite sequencing of CpG islands 3 and 7 was performed in 21 and 23 CLL patients respectively and in five healthy controls. A representative electropherogram depicting two methylated (C) or unmethylated (T) CpG sites in island 3 located in 5’ region of *PAX9* is shown

Genomic DNA (2 μg) was bisulfite modified and purified using Epitect Bisulfite kit as per the manufacturer’s instructions (Qiagen, Hilden , Germany). The bisulfite converted DNA was amplified for two CpG islands in *PAX9* gene as depicted in Fig. 1 and sequenced with BigDye Terminator v3.1 Cycle Sequencing kit (Applied Biosystems, CA, USA) with primers designed using MethPrimer (http://www.urogene.org/cgi-bin/methprimer/methprimer.cgi). The percent methylation levels were computed and further analysed with Bisulfite Sequencing DNA Methylation Analysis (BISMA) software (http://services.ibc.uni-stuttgart.de/BDPC/BISMA/).

### Real-time quantitative PCR (RQ-PCR)

The mRNA expression based microarray findings were validated using RQ-PCR in an independant cohort of 93 early stage CLL patients for 17 genes with gene-specific primers (Additional file 1: Table S1). The experiments were performed using SYBR Green Master Mix according to the manufacturer’s protocol on Mx3005P (Agilent Technologies, Santa Clara, CA, USA). The fold change was calculated using 2^-ΔΔCt^ method *with beta-actin* as an endogenous control. The Receiver’s operating characteristic (ROC) curve-derived cut-off values were used to define high or low mRNA expression levels.

### Bioinformatics analysis and statistics

Methylation array data was analyzed using Genomic Workbench version 7.0 (Agilent Technologies, Santa Clara, CA, USA). On the basis of melt temperature, log-ratio data for each probe was normalized. By taking into account the Gaussian-fit curves, Z score was generated for each sample and *p* values were calculated. The *p* values were then used to determine the log-odds score for each probe. The differentially hypermethylated and hypomethylated probes between groups were filtered based on the minimum value of log2-fold change (log2FC) between the groups =0.25, *p* < 0.05 and the false discovery rates (FDR) of 0.2 [24].The probes with log2FC ≤ (-)0.25 were considered hypomethylated and ≥ (+)0.25 were considered hypermethylated.

The gene expression data across all arrays was log2 transformed and normalized using quantile normalization and analyzed by the Lima library from R-Bioconductor. Probes with an adjusted p-value less than 0.05 and log2FC of 1 were selected.

The correlation of log-odds values obtained from the DNA methylation arrays (*p* < 0.05, log2FC = 0.25) and the expression arrays for the identified genes was used as an indicator of the correlation between DNA methylation and gene expression. The probes showing hypomethylation (log2FC ≤ (-)0.25, *p* < 0.05) in conjunction with higher expression (log2 FC > 1, *p* < 0.05) between any two compared conditions were identified. Similarly, the probes exhibiting hypermethylation (log2FC ≥ 0.25, *p* < 0.05) in conjunction with lower expression (log2 FC < (-)1, *p* < 0.05) were also identified.

Receiver’s operating characteristic curve was used to calculate the cut-off value to determine the low and high expression of a particular gene. The differences in mRNA expression between the groups as obtained from RQ-PCR were compared using the Mann-Whitney Rank Sum test or Kruskal-Wallis One Way Analysis of Variance on Ranks. The relative risk (RR) of treatment initiation was assessed using the Chi-square statistic with Yate’s continuity correction. The time to first treatment (TTFT) and overall survival (OS) were compared between the groups using the Kaplan-Meier survival analysis followed by the log-rank test. Hazard ratio (HR) for each variable was calculated using the Cox proportional hazard regression (Sigma Plot Version 13.0, Systat Software, Inc.).

### Data access

The DNA methylation as well as the mRNA expression data generated in the study have been submitted to the NCBI Gene Expression Omnibus (GEO) (http://www.ncbi.nlm.nih.gov/geo/) under accession number GSE81937.

## Results

### Methylation profile

**Fig. 2:**
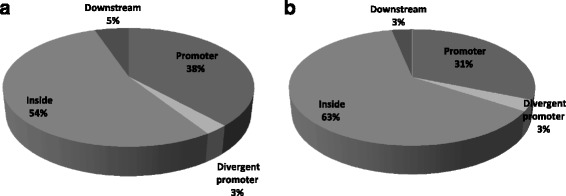
Distribution of **a** hypermethylated and **b** hypomethylated probes in CLL *Vs.* CD19+ normal controls

A comparison of differential methylation between CLL (*n* = 14) and normal CD19+ B-cells identified a total of 6129 probes to be differentially methylated which were further classified as hypermethylated (5254 probes, 2505 genes) or hypomethylated (875 probes, 753 genes). The differentially methylated probes that represented unknown genes, non-coding RNAs, hypothetical proteins, chromosomal loci, predicted open reading frames, and probes associated with sex-chromosomes were excluded from the downstream analysis. Among the differentially methylated probes, 53.8% of hypermethylated probes were located inside known gene bodies, 38.2% in the promoters, 2.6% in divergent promoters and 5.2% were located downstream of the known genes (Fig. 2a). The frequency distribution of the hypomethylated probes (Fig. 2b) was comparable to the hypermethylated probes. Of the differentially methylated probes, CpG sites were found in 73% of the hypermethylated probes and in 81% of the hypomethylated probes. The details pertaining to these probes, including the gene name, chromosomal location and distribution are provided in Additional file 1: Tables S2A and S2B.

On the basis of gene functions, the CpG islands in the promoter regions of the tumor suppressor genes (*KLF4*, *PTCH1*, *PAX5*, *PCDH10*, *RASSF10*, *IRX1*, *TBX5*, *ID4*, *SOX7*, *SLIT2*) and the transcription factors (*TWIST1*, *KLF4*, *TAL1*, *PAX2*, *PAX9*, *NR2F2*, *IRX4*, *MEIS1)* were found to be hypermethylated. Approximately, 10% of the hypermethylated CpG promoters were located within the homeobox genes. Promoter regions of genes such as *FOXD3*, *FOXE1*, *FOXG1*, *ID4*, *SLIT2*, *BNC1*, *SALL1*, *RIPK4*, *HAND2*, *SOX9*, *SOX11*, *NR2F2*, *TAL1*, *SIM2*, *PAX9*, and *TBX2* were also found to be hypermethylated in sync with earlier reported results in CLL [16, 19, 25]. In addition, hypomethylation was observed in the promoter region of *NFATC1* and inside gene body in *NOTCH1*, *SFRP1*, and *GPS* as has been reported in earlier studies in CLL [19, 25]. Using the Database for Annotation, Visualization and Integrated Discovery (DAVID) functional analysis tool, the differentially methylated genes were evaluated for the overrepresented Gene Ontology (GO) categories and the most significant overrepresented GO biological processes were found to be related to regulation of transcription (*p* < 0.0001) [26, 27].

To identify the association of differential methylation profile with the *IGHV* mutation status, the methylation array data from 9 *IGHV* unmutated and 5 *IGHV* mutated cases was compared. This analysis elicited a distinct signature of 56 hypermethylated (*p* < 0.05, log2FC ≥ 0.25) and 2402 hypomethylated probes (*p* < 0.05, log2FC ≤ (-)0.25) in unmutated CLL. The hypermethylated probes were distributed across 46 genes and spanned promoter regions of 10 genes (Additional file 1: Table S3A).Similarly, the hypomethylated probes spread across 1332 genes and spanned promoter regions of 399 genes (Additional file 1: Table S3B). Differential methylation of several genes previously reported in the *IGHV* mutation based subgroups [*NCOR2*, *KCNJ2*, *SIX3*, *CHRM1*, [16]], [*NRF1*, *CRY1*, *KCNJ2*, *SOX5* [28]] was also noticed in the present study. In addition, differential CpG promoter hypomethylation of genes already known to influence clinical outcome in other malignancies was observed and includes *EMILIN2* [29], *TBX5* [30], *CBX8* [31],*OLIG2* [32], and *PCDH10* [33]. The DAVID database was used to identify biological pathways for the differentially methylated genes. Four of the Kyoto Encyclopedia of Genes and Genomes (KEGG) pathways including circadian rhythm pathway (*p* = 0.002), calcium signalling pathway (*p* = 0.03), axon guidance (*p* = 0.02), and gap junction pathway (*p* = 0.04) were found to be significantly affected in the *IGHV* unmutated Vs*.* mutated subgroup.

### Correlation of methylation and gene expression analysis

To investigate the possible influence of CpG methylation status on the expression level of the corresponding genes, the gene expression profiles were integrated with the DNA methylation profiles and co-analyzed. On comparing the data of CLL patients with healthy individuals, a negative correlation in CpG methylation and gene expression was observed for 211genes (Additional file 1: Table S4). Of these, 149 genes were hypermethylated and downregulated and 62 genes were hypomethylated and upregulated including *AXIN2*, *ID4*, *EBF1*, *SOX4*, *SOX7*, *TAL1*, *PMEPA1*, *SPRY1*, *CDK6*, and *MEIS1*. Pathway analysis using the genes having negative correlation for DNA methylation and gene expression in CLL Vs. normal CD19+ cells identified significant enrichment of three KEGG pathways which included *p53* signalling pathway (*p* = 0.002), pathways in cancer (*p* = 0.005), and the cell cycle pathway (*p* = 0.007).

**Fig. 3:**
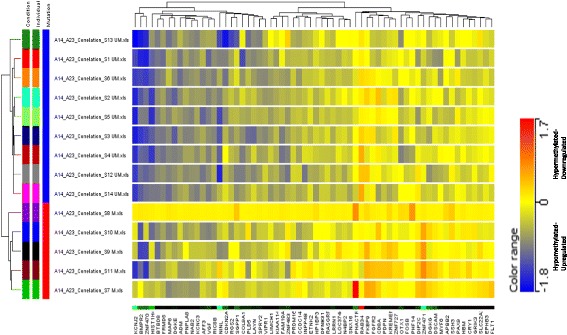
Supervised hierarchical clustering of hypermethylated-dowregulated and hypomethylated -upregulated genes selected on the basis of significant log2FC values in *IGHV* mutated (*n* = 05) Vs*. IGHV* unmutated (*n* = 09) CLL. The Euclidean hierarchical clustering was performed using Gene Spring Gx software version 13.5 and is based on normalized intensity values. Each *row* represents an individual patient and each *column* represents a gene. A gradient color scale ranging between *blue* (hypermethylated and downregulated) and *red* (hypomethylated and upregulated) is included

A comparison of the CpG methylation and gene expression profiles among the *IGHV* unmutated *Vs*. mutated patients identified 64 differentially expressed genes (Fig. 3) including *BMPR2*, *CRY1*, *FGFR2*, *FOSB*, *INPP4B*, *PLD5*, *PAX9*, *RGS2*, *RIC8B*, and *VIPR1* (Additional file 1: Table S5).

**Fig. 4:**
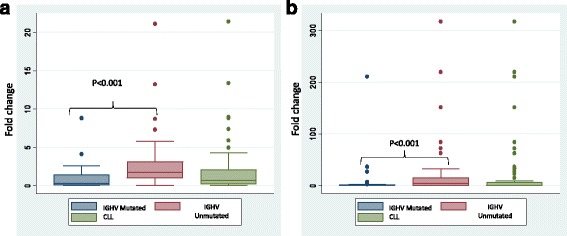
Box-plot representation of mRNA expression fold change as assessed by RQ-PCR for **a** *CRY1* gene in CLL and its *IGHV* mutated and unmutated subgroups **b** *PAX9* gene in CLL and its *IGHV* mutated and unmutated subgroups. Box-plot graphs show median (*middle line*), interquartile range (*box*), 25–75th percentile (whiskers) and statistically significant difference (*p* value) estimated in comparison between different groups

Of the various genes found to be differentially methylated and/or differentially expressed, a total of 17 genes (Table 2) were validated using RQ-PCR on a cohort of 93 (22 female: 71 male) early stage CLL patients and pooled CD19+ B cells from 10 healthy volunteers. The criteria for selection of these genes was negative correlation between CpG promoter methylation and gene expression in CLL Vs. normal (*MEIS1*, *PMEPA1*, *SOX7*, *SPRY1*, *CDK6*, *ID4*, *AXIN2*, *TNRC18*) and in the *IGHV* unmutated *Vs.* mutated subgroup (*CRY1*, *VIPR1*, *PAX9*, *RIC8B*). Other genes selected for validation included *NFATC1* (hypomethylated in CLL), *TBX2*, *TSHZ3* (hypermethylated in CLL), *SPRY2* (upregulated in CLL) and *BIK* (downregulated in CLL). We focused on these five genes as they had previously been shown in the literature to be epigenetically influenced in CLL [*NFATC1* [11]], or in other malignancies [*BIK* [34], *SPRY2* [35], *TBX2* [36, 37], *TSHZ3* [38, 39]]. As expected, *MEIS1, PMEPA1, SOX7, SPRY1, CDK6, TBX2* were significantly downregulated (*p* < 0.05) while *SPRY2* (*p* = 0.016), *VIPR*1 (*p* = 0.04) and *ID4* (*p* = 0.03) were significantly upregulated in CLL cells as compared to healthy B-cells. Though not significant, *AXIN2* was upregulated and *TNRC18, NFATC1* and *BIK* were downregulated in CLL as compared to healthy CD19+ cells (Table 2). The expression of only *CRY1* and *PAX9* differed significantly (*p* < 0.05) with respect to the *IGHV* mutation status (Table 2, Fig. 4)

**Table 2:** Comparison of mRNA levels of selected genes (median ∆Cq and median fold change) as assessed by real time RQ-PCR in CLL (*n* = 93; unmutated = 39; mutated = 54) and CD19+ sorted B-cells from healthy individuals (*n* = 10)

Comparison of levels of mRNA expression of selected genes in CLL									
S.No.	Gene	CLL Vs. normal				Mutated CLL Vs. unmutated CLL			
		Group	Median ∆Cq	Median fold change	*p* value	IGHV Mutation status	Median ∆Cq	Median fold change	*p* value
1.	CRY1	CLL	8.61	0.75	0.91	Mutated	9.8	0.31	*<0.001*
		19+ Normal	8.29			Unmutated	7.46	1.73	
2.	MEIS1	CLL	13.03	0.07	*0.01*	Mutated	12.85	0.05	0.85
		19+ Normal	8.87			Unmutated	12.63	0.06	
3.	ID4	CLL	13.75	4.37	*0.03*	Mutated	13.51	3.89	0.45
		19+ Normal	15.7			Unmutated	13.71	3.2	
4.	TNRC18	CLL	13.21	0.64	0.43	Mutated	12.93	0.62	0.6
		19+ Normal	12.53			Unmutated	13.34	0.59	
5.	NFATC1	CLL	8.43	0.5	0.47	Mutated	8.86	0.39	0.37
		19+ Normal	7.54			Unmutated	8.37	0.57	
6.	CDK6	CLL	12.65	0.2	*0.02*	Mutated	12.65	0.25	0.7
		19+ Normal	10.48			Unmutated	12.65	0.26	
7.	VIPR1	CLL	6.62	7.9	*0.04*	Mutated	6.5	6.96	0.32
		19+ Normal	9.34			Unmutated	5.98	11.4	
8.	SPRY1	CLL	12.75	0.04	*<0.001*	Mutated	12.18	0.05	0.28
		19+ Normal	8.49			Unmutated	12.7	0.03	
9.	PAX9	CLL	12.29	0.81	0.66	Mutated	12.8	0.37	*<0.001*
		19+ Normal	11.61			Unmutated	9.28	4.61	
10.	PMEPA	CLL	12.33	0.01	*<0.001*	Mutated	12.32	0.01	0.47
		19+ Normal	5.52			Unmutated	11.72	0.01	
11.	TBX2	CLL	16.92	0.1	*0.004*	Mutated	16.42	0.08	0.67
		19+ Normal	13.45			Unmutated	16.91	0.08	
12.	TSHZ3	CLL	10.9	1.19	0.81	Mutated	10.57	1.15	0.56
		19+ Normal	10.97			Unmutated	11.18	0.82	
13.	BIK	CLL	8.27	0.4	0.5	Mutated	8.4	0.44	0.54
		19+ Normal	7.26			Unmutated	8.08	0.53	
14.	SPRY2	CLL	10.2	6.2	*0.02*	Mutated	9.78	6.59	0.42
		19+ Normal	12.4			Unmutated	10.26	4.6	
15.	AXIN2	CLL	9.8	6.3	0.23	Mutated	9.2	4.79	0.56
		19+ Normal	11.77			Unmutated	8.91	6.66	
16.	SOX7	CLL	11.93	0.18	*0.02*	Mutated	11.65	0.18	0.99
		19+ Normal	9.23			Unmutated	11.92	0.16	
17.	RIC8B	CLL	8.77	1.85	0.38	Mutated	8.59	1.85	0.78
		19+ Normal	9.61			Unmutated	8.72	1.75	

The statistically significant *p* values are shown in italics

The status of hypomethylation of *PAX9* among unmutated CLL was confirmed through bisulfite genome sequencing of CpG island 3 in close proximity to CpG110 (Fig. 1).While CpG island 7 did not reveal any significant difference in methylation levels, the average % methylation at CpG island 3 was found to be 52.74% in mutated while 24.72% among unmutated CLL. This further corroborates with the reduced expression of *PAX9* in unmutated group of CLL patients as established through microarray-based observations.

### Association between gene expression and clinical outcome

**Fig. 5:**
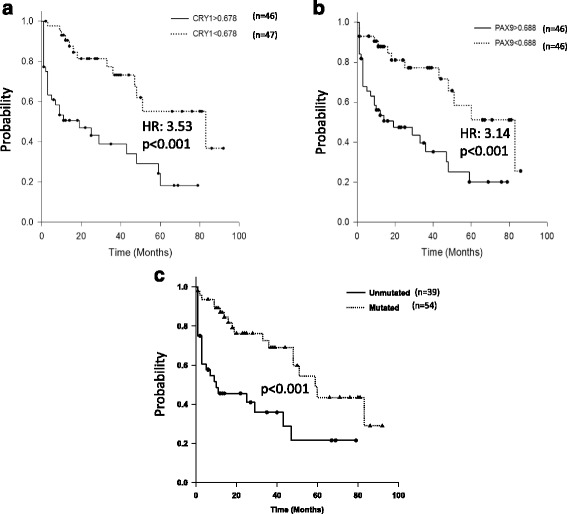
Kaplan-Meier survival curves representing time to first treatment (TTFT) in early stage CLL patients with **a** low (*n* = 47) and high (*n* = 46) mRNA expression of *CRY1* **b** low (*n* = 46) and high (*n* = 46) mRNA expression of *PAX9* and; **c** unmutated (*n* = 39) and mutated *IGHV* genes (*n* = 54)

**Fig. 6:**
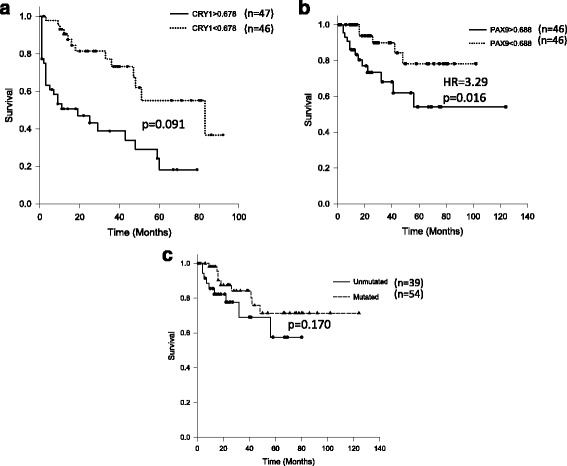
Kaplan-Meier survival curves representing overall survival in early stage CLL patients with **a** low (*n* = 47) and high (*n* = 46) mRNA expression of *CRY1* **b** low (*n* = 46) and high (*n* = 46) mRNA expression of *PAX9* and; **c** unmutated (*n* = 39) and mutated *IGHV* genes (*n* = 54)

Of the 17 genes evaluated for mRNA expression, *CRY1* (*p* = 0.008) and *PAX9* (*p* < 0.001) were expressed at higher levels in Rai stage I and II as compared to stage 0. A progressive increase in the expression of *CRY1* (*p* = 0.004) and *PAX9* (*p* < 0.001) was observed in increasing IPI score categories ranging from 1 to 4. We further explored the association between expression level of candidate genes with relative risk of treatment initiation, TTFT and OS (Table 3). The relative risk of treatment initiation was significantly higher with high expression of *PAX9* (*p* = 0.001) or *CRY1* (*p* = 0.005). The high expressions of both *PAX9* (HR 3.14, 95% CI 1.589–6.205, *p* < 0.001) as well as *CRY1* (HR 3.53, 95% CI 1.789–6.987, *p* < 0.001) were significantly associated with shorter TTFT (Fig. 5). However, high expression of only *PAX9* gene (HR 3.29, 95% CI 1.172–9.272, *p* = 0.016) was significantly associated with shorter OS (Fig. 6).

**Table 3:** Association of relative risk of treatment initiation, time to first treatment and overall survival with the mRNA expression of 17 genes selected for validation in early stage CLL patients (*n* = 93)

Association of RR, TTFT and OS with mRNA expression																	
S No.	Parameter (cut off)		Treatment required		RR of treatment initiation			TTFT (in months)					OS (in months)				
			Yes	No	RR	95% CI	*p* value	Time	Events	HR	95% CI	p value	Time	Events	HR	95% CI	*p* value
1	CRY1 (0.678)	<0.678	16	31	1.91	1.221-3.006	*0.01*	83	13				NR	7			
		>0.678	30	16				19	28	3.53	1.789-6.987	*<0.001*	NR	11	2.22	0.857-5.754	0.1
2	MEIS1 (0.057)	<0.057	23	23	0.97	0.64-1.47	0.92	48	21				NR	9			
		>0.057	23	24				47	22	1.04	0.564-1.925	0.9	NR	9	1.06	0.421-2.707	0.89
3	ID4 (3.891)	<3.891	20	25	1.21	0.80-1.85	0.47	51	18				NR	11			
		>3.891	26	22				47	23	1.06	0.571-1.975	0.85	NR	7	1.85	0.654-5.245	0.25
4	TNRC18 (0.646)	<0.646	24	22	0.89	0.59-1.35	0.76	43	22				NR	11			
		>0.646	22	25				48	19	0.86	0.465-1.596	0.63	NR	7	0.64	0.248-1.657	0.36
5	NFATC1 (0.536)	<0.536	23	23	0.97	0.64-1.47	0.92	48	20				NR	7			
		>0.536	23	24				36	21	1.45	0.775-2.712	0.25	NR	11	0.95	0.364-2.503	0.93
6	CDK6 (0.247)	<0.247	22	23	1.02	0.67-1.54	0.92	48	21				NR	7			
		>0.247	24	24				47	22	1.05	0.570-1.945	0.87	NR	11	1.75	0.677-4.542	0.26
7	VIPR1 (7.21)	<7.210	19	26	1.33	0.87-2.03	0.25	48	17				NR	10			
		>7.210	27	21				36	24	1.6	0.857-2.997	0.14	NR	8	0.98	0.384-2.508	0.97
8	SPRY1 (0.08)	<0.080	24	25	0.91	0.60-1.38	0.84	51	16				NR	11			
		>0.080	22	24				29	25	1.77	0.949-3.335	0.07	NR	7	2.13	0.797-5.714	0.13
9	PAX9 (0.688)	<0.688	16	30	1.87	1.19-2.93	*0.001*	83	13				NR	5			
		>0.688	30	16				19	28	3.14	1.589-6.205	*<0.001*	NR	13	3.29	1.172-9.272	*0.02*
10	PMEPA1 (0.01)	<0.010	19	27	1.42	0.93-2.16	0.14	51	17				NR	5			
		>0.010	27	19				43	24	1.34	0.723-2.518	0.34	NR	13	1.97	0.698-5.565	0.2
11	TBX2 (0.085)	<0.085	24	21	0.87	0.583-1.322	0.68	48	21				NR	8			
		>0.085	22	25				47	22	0.96	0.523-1.792	0.92	NR	10	1.44	0.567-3.655	0.44
12	TSHZ3 (1.073)	<1.073	25	21	0.84	0.556-1.268	0.52	47	20				NR	8			
		>1.073	21	25				48	21	0.94	0.510-1.740	0.85	NR	10	1.38	0.546-3.511	0.49
13	BIK (0.438)	<0.438	19	26	1.33	0.87-2.04	0.25	43	16				NR	9			
		>0.438	26	20				47	24	1.23	0.652-2.330	0.52	NR	9	0.8	0.319-2.052	0.66
14	SPRY2 (5.464)	<5.464	22	23	1.02	0.67-1.55	0.92	48	19				NR	10			
		>5.464	23	23				48	21	0.86	0.459-1.614	0.64	NR	8	0.6	0.237-1.537	0.29
15	AXIN2 (5.979)	<5.979	23	22	0.93	0.61-1.41	0.92	29	21				NR	8			
		>5.979	22	24				59	19	0.73	0.393-1.375	0.34	NR	10	1.06	0.418-2.695	0.9
16	SOX7 (0.162)	<0.162	20	25	1.22	0.80-1.86	0.46	48	18				NR	10			
		>0.162	25	21				47	22	1.15	0.620-2.163	0.64	NR	8	0.68	0.270-1.744	0.43
17	RIC8B (1.741)	<1.741	19	24	1.22	0.80-1.87	0.46	48	17				NR	10			
		>1.741	26	22				43	23	1.08	0.577-2.028	0.81	NR	8	0.57	0.226-1.463	0.25

Abbreviations used: *RR* relative risk, *TTFT* time to first treatment, *OS* overall survival, *NR* not reached, *HR* hazard ratio

The statistically significant *p* values are shown in italics

## Discussion

Extremely variable clinical course of early stage CLL patients highlights the importance of well-described prognostic markers for clinical management of these patients. Various prognostic markers that are currently in use include *IGHV* mutational status [2], genomic abnormalities [3], expression of *ZAP-70* [4], and CD38 [2]. Recent studies have associated specific DNA methylation signatures with specific prognostic subgroups in CLL [14–16, 19]. The present study has dealt with the methylation profiling of early stage CLL patients on the basis of their *IGHV* mutational status.

The study has identified differential methylation of several genes such as *NCOR2*, *SIX3*, *CHRM1*, *NRF1*, *CRY1*, *KCNJ2*, and *SOX5* that have been reported earlier to be differentially methylated in the *IGHV*-gene based subgroups [16, 28]. Besides, an association of promoter hypomethylation of *MYLK* with the *IGHV* unmutated cases was also observed in the current study. Since, higher expression of *MYLK* is known to be significantly correlated with poor clinical outcome [40], it is plausible that promoter hypomethylation of *MYLK* in the *IGHV* unmutated cases might be associated with poor prognosis. Furthermore, differential CpG promoter hypomethylation of two important hematopoietic transcription factors *MEIS1* and *TAL1* which are known methylation targets in B-cell ALL was also observed [41].

An analysis of signalling pathway network for genes with perturbed methylation profiles observed among *IGHV* unmutated patients indicated the involvement of calcium signalling pathway. Previous studies have suggested that altered Ca^2+^ signalling contributes to major tumor progression events including proliferation, migration, invasion, and metastasis [42, 43]. Recently, Muggen et al. [44] demonstrated an association of the *IGHV* mutational status with the level of basal Ca^2+^ signalling in CLL. The present study provides evidence that aberrant methylation of genes involved in the calcium signalling pathway might be one of the mechanisms responsible for net differences in the basal Ca^2+^ signalling events.

In the present analysis, an inverse correlation between methylation and gene expression was observed for 209 genes in CLL including transcription factors (*ID4, NFATC1, TBX2, TAL1, MEIS1)*, *SPRY* family members (*SPRY1*, *SPRY2)* and *SOX* family members (*SOX4*, *SOX7)*. Correlation of promoter methylation of *ID4* gene with shortened patient survival has been already documented in CLL [45]. An association of methylation of *TBX2* [37] and *SPRY2* [35] with disease progression has also been demonstrated in bladder cancer and B-cell diffuse lymphoma, respectively, but so far, no studies have been reported in CLL.

Screening of inversely correlated genes associated with *IGHV* mutation status revealed only 21 gene promoters to be significantly hypomethylated and upregulated in unmutated cases (Additional file 1: Table S5). One of these genes encodes for bone morphogenetic protein (*BMP*) receptor II which is a serine/threonine receptor kinase and has previously been shown to be involved in molecular pathogenesis of hematological malignancies including acute myelomonocytic leukemia, acute promyelocytic leukemia, multiple myeloma as well as CLL [46, 47]. Cell surface expression of BMP receptors (BMPRIA and BMPRIB) have been shown to be elevated in advanced stages of CLL [47]. In-vitro studies have shown that co-expression of BMPRII facilitates BMP binding to its receptors and therefore contributes to downstream biological functions [48, 49]. This is in line with the results of the present study wherein upregulated *BMPRII* gene expression and hypomethylation of *BMPRII* gene was noticed among unmutated subgroup of CLL patients.

Alterations in methylation status and associated gene expression levels of another gene *CRY1* have also been reported in prognostically distinct subsets of CLL [50] as well as in CML [51]. Our study confirms the possible influence of hypomethylation and upregulated expression of *CRY1* in prognostically poor *IGHV* unmutated CLL and further emphasises its role as potential biomarker for relative risk of treatment initiation and TTFT in early stage CLL. In addition to *CRY1*, three other circadian rhythm genes *NPAS2*, *BHLHE40*, and *ARNTL* were also observed to be hypomethylated in the unmutated subgroup [52].

*PAX9* is one of the nine members of "paired box” (*PAX*)-containing transcription factor family and its inhibition has been shown to induce apoptosis with increased cleavage of caspase-3 and *PARP*, increased expression of *BAX* and decreased expression of *BCL-2* in oral squamous cell carcinoma [53]. In the recent years, it has emerged as one of the biomarkers of cell proliferation in lung cancer [54]. A significant association of *PAX9* expression with stage, IPI score, relative risk of treatment initiation, TTFT and OS in the present study strengthens its role as an important marker of prognosis in CLL as well.

Since levels of expression of either *PAX9* or *CRY1* did not show significant difference in CLL patients when compared to healthy controls but rather between patients subgrouped on the basis of the *IGHV* mutational status, it is plausible that these two genes may be involved in progression of CLL rather than development of the disease. This explanation is further supported by progressively increasing gene expression levels of *PAX9* and *CRY1* in coherence with advanced Rai stage and higher IPI scores. The mechanism(s) underlying such an influence of these two genes in CLL pathogenesis are not known but might involve apoptotic [53, 55–57], or analogous pathways involved in cancer.

Besides, several aberrantly methylated genes were also identified in *IGHV* mutational status based subgroups which could serve as potential markers in CLL. The major limitation of the present study was that a limited number of genes were evaluated in a small cohort of early stage CLL patients. Further studies on large cohorts of early stage CLL patients for expression patterns of additional set of genes are required that may help in characterizing the functional role of the genes identified in the present study. Identification of relevant epigenetically influenced genes that have an impact on gene expression as well as clinical outcome may pave way for identification and development of therapeutically relevant drug targets.

## Conclusions

The present study confirms the prognostic role of *CRY1* in CLL as its aberrant methylation and expression is associated with high risk of treatment initiation and shorter time to first treatment. In addition, this study highlights *PAX9* as a novel marker of prognostication in CLL as its expression was significantly associated with high risk of treatment initiation, shorter time to first treatment and overall survival.

## Additional file

Additional file 1: Table S1. List of primers used in RQ-PCR studies. **Table S2A.** List of probes hypermethylated in CLL in comparison to CD19+ cells from healthy individuals. **Table S2B.** List of probes hypomethylated in CLL in comparison to normal CD19+ cells. **Table S3A.** List of probes hypermethylated in unmutated in comparison to mutated CLL. **Table S3B.** List of probes hypomethylated in unmutated CLL in comparison to mutated CLL. **Table S4.** List of genes having negative correlation for methylation and gene expression in CLL as compared to normal 19+ cells. **Table S5.** List of genes having negative correlation for methylation and gene expression in unmutated CLL as compared to mutated CLL. (XLS 1339 kb)

## References

[1] Chiorazzi N, Rai KR, Ferrarini M (2005). Chronic lymphocytic leukemia. N Engl J Med.

[2] Damle RN, Wasil T, Fais F, Ghiotto F, Valetto A, Allen SL (1999). IgV gene mutation status and CD38 expression as novel prognostic indicators in chronic lymphocytic leukemia. Blood.

[3] Krober A, Seiler T, Benner A, Bullinger L, Brückle E, Lichter P (2002). V (H) mutation status, CD38 expression level, genomic aberrations, and survival in chronic lymphocytic leukemia. Blood.

[4] Dürig J, Nückel H, Cremer M, Führer A, Halfmeyer K, Fandrey J (2003). ZAP-70 expression is a prognostic factor in chronic lymphocytic leukemia. Leukemia.

[5] Gentile M, Mauro FR, Calabrese E, De Propris MS, Giammartini E, Mancini F (2005). The prognostic value of CD38 expression in chronic lymphocytic leukemia patients studied prospectively at diagnosis: a single institute experience. Br J Haematol.

[6] Eisele L, Haddad T, Sellmann L, Dührsen U, Dürig J (2009). Expression levels of CD38 on leukemic B cells but not on non-leukemic T cells are comparably stable over time and predict the course of disease in patients with chronic lymphocytic leukemia. Leuk Res.

[7] Wahlfors J, Hiltunen H, Heinonen K, Hämäläinen E, Alhonen L, Jänne J (1992). Genomic hypomethylation in human chronic lymphocytic leukemia. Blood.

[8] Rush LJ, Raval A, Funchain P, Johnson AJ, Smith L, Lucas DM (2004). Epigenetic profiling in chronic lymphocytic leukemia reveals novel methylation targets. Cancer Res.

[9] Raval A, Lucas DM, Matkovic JJ, Bennett KL, Liyanarachchi S, Young DC (2005). TWIST2 demonstrates differential methylation in immunoglobulin variable heavy chain mutated and unmutated chronic lymphocytic leukemia. J Clin Oncol.

[10] Raval A, Byrd JC, Plass C (2006). Epigenetics in chronic lymphocytic leukemia. Semin Oncol.

[11] Liu TH, Raval A, Chen SS, Matkovic JJ, Byrd JC, Plass C (2006). CpG island methylation and expression of the secreted frizzled-related protein gene family in chronic lymphocytic leukemia. Cancer Res.

[12] Corcoran M, Parker A, Orchard J, Davis Z, Wirtz M, Schmitz OJ (2005). ZAP-70 methylation status is associated with ZAP-70 expression status in chronic lymphocytic leukemia. Haematologica.

[13] Strathdee G, Sim A, Parker A, Oscier D, Brown R (2006). Promoter hypermethylation silences expression of the HoxA4 gene and correlates with IgVh mutational status in CLL. Leukemia.

[14] Kanduri M, Cahill N, Goransson H, Enström C, Ryan F, Isaksson A (2010). Differential genome-wide array-based methylation profiles in prognostic subsets of chronic lymphocytic leukemia. Blood.

[15] Cahill N, Bergh AC, Kanduri M, Göransson-Kultima H, Mansouri L, Isaksson A (2013). 450 K-array analysis of chronic lymphocytic leukemia cells reveals global DNA methylation to be relatively stable over time and similar in resting and proliferative compartments. Leukemia.

[16] Ronchetti D, Tuana G, Rinaldi A, Agnelli L, Cutrona G, Mosca L (2014). Distinct patterns of global promoter methylation in early stage chronic lymphocytic leukemia. Genes Chromosomes Cancer.

[17] Rahmatpanah FB, Carstens S, Hooshmand SI, Welsh EC, Sjahputera O, Taylor KH (2009). Large-scale analysis of DNA methylation in chronic lymphocytic leukemia. Epigenomics.

[18] Kanduri M, Marincevic M, Halldórsdóttir AM, Mansouri L, Junevik K, Ntoufa S (2012). Distinct transcriptional control in major immunogenetic subsets of chronic lymphocytic leukemia exhibiting subset-biased global DNA methylation profiles. Epigenetics.

[19] Tong WG, Wierda WG, Lin E, Kuang SQ, Bekele BN, Estrov Z (2010). Genome-wide DNA methylation profiling of chronic lymphocytic leukemia allows identification of epigenetically repressed molecular pathways with clinical impact. Epigenetics.

[20] Jones PA (2012). Functions of DNA methylation: islands, start sites, gene bodies and beyond. Nat Rev Genet.

[21] Rai K, Gale R, Rai K (1987). A critical analysis of staging in CLL. Chronic Lymphocyte Leukemia: Recent progress and future direction.

[22] Kutsch N, Bahlo J, Byrd J C, Dohner H, Eichhorst B, Else M, et al. The international prognostic index for patients with CLL (CLL-IPI): An international meta-analysis. Journal of Clinical Oncology, ASCO Annual Meeting Abstracts. 2015; 33, suppl; abstr 7002.

[23] van Dongen JJ, Langerak AW, Brüggemann M, Evans PA, Hummel M, Lavender FL (2003). Design and standardization of PCR primers and protocols for detection of clonal immunoglobulin and T-cell receptor gene recombinations in suspect lymphoproliferations: report of the BIOMED-2 Concerted Action BMH4-CT98-3936. Leukemia.

[24] Provençal N, Suderman MJ, Guillemin C, Vitaro F, Côté SM, Hallett M (2014). Association of childhood chronic physical aggression with a DNA methylation signature in adult human T- cells. PLoS One.

[25] Pei L, Choi J, Liu J, Lee E, McCarthy B, Wilson JM (2012). Genome-wide DNA methylation analysis reveals novel epigenetic changes in chronic lymphocytic leukemia. Epigenetics.

[26] da Huang W, Sherman BT, Lempicki RA (2009). Systematic and integrative analysis of large gene lists using DAVID bioinformatics resource. Nat Protoc.

[27] da Huang W, Sherman BT, Lempicki RA (2009). Bioinformatics enrichment tools: paths toward the comprehensive functional analysis of large gene lists. Nucleic Acids Res.

[28] Kulis M, Heath S, Bibikova M, Queirós A, Navarro A, Clot G (2012). Epigenomic analysis detects widespread gene-body DNA hypomethylation in chronic lymphocytic leukemia. Nat Genet.

[29] Hill V, Hesson LB, Dansranjavin T, Dallol A, Bieche I, Vacher S (2010). Identification of 5 novel genes methylated in breast and other epithelial cancers. Mol Cancer.

[30] Yu J, Ma X, Cheung KF, Li X, Tian L, Wang S (2010). Epigenetic inactivation of T-box transcription factor 5, a novel tumor suppressor gene, is associated with colon cancer. Oncogene.

[31] Xiao W, Ou C, Qin J, Xing F, Sun Y, Li Z (2014). CBX8, a novel DNA repair protein, promotes tumorigenesis in human esophageal carcinoma. Int J ClinExp Pathol.

[32] Teneng I, Tellez CS, Picchi MA, Klinge DM, Yingling CM, Snider AM (2015). Global identification of genes targeted by DNMT3b for epigenetic silencing in lung cancer. Oncogene.

[33] Narayan G, Freddy A, Xie D, Liyanage H, Clark L, Kisselev S (2011). Promoter methylation-mediated inactivation of PCDH10 in acute lymphoblastic leukemia contributes to chemotherapy resistance. Genes Chromosomes Cancer.

[34] Hatzimichael E, Dasoula A, Dranitsaris G, Vassou A, Papoudou-Bai A, Stebbing J, et al. BIK (Bcl2-Interacting Killer) CpG methylation status as a potential predictive biomarker of relapsed/refractory multiple myeloma disease. Journal of Clinical Oncology, ASCO Annual Meeting Abstracts.2010; Vol 28, No 15 suppl (May 20 Supplement), 8118.

[35] Sanchez A, Setien F, Martınez N, Oliva JL, Herranz M, Fraga MF (2008). Epigenetic inactivation of the ERK inhibitor Spry2 in B-cell diffuse lymphomas. Oncogene.

[36] Kandimalla R, van Tilborg AA, Kompier LC, Stumpel DJ, Stam RW, Bangma CH (2012). Genome-wide analysis of CpG island methylation in bladder cancer identified TBX2, TBX3, GATA2, and ZIC4 as pTa-Specific prognostic markers. Eur Urol.

[37] Beukers W, Kandimalla R, Masius R, Vermeij M, Kranse R, Leenders G (2015). Stratification based on methylation of TBX2 and TBX3 into three molecular grades predicts progression in patients with pTa-bladder cancer. Mod Pathol.

[38] Yamamoto M, Cid E, Bru S, Yamamoto F (2011). Rare and frequent promoter methylation, respectively, of TSHZ2 and 3 genes that are both downregulated in expression in breast and prostate cancers. PLoS One.

[39] Dunwell T, Hesson L, Rauch TA, Wang L, Clark RE, Dallol A (2010). A genome-wide screen identifies frequently methylated genes in haematological and epithelial cancers. Mol Cancer.

[40] Shukla A, Chaturvedi N, Ahrens A, Cutucache C, Mittal A, Bierman P (2013). Stromal tumor microenvironment in chronic lymphocytic leukemia: regulation of leukemic progression. J Leuk (Los Angel).

[41] Musialik E, Bujko M, Kober P, Wypych A, Gawle-Krawczyk K, Matysiak M (2015). Promoter methylation and expression levels of selected hematopoietic genes in pediatric B-cell acute lymphoblastic leukemia. Blood Res.

[42] Monteith GR, McAndrew D, Faddy HM, Roberts-Thomson SJ (2007). Calcium and cancer: targeting Ca^2+^ transport. Nat Rev Cancer.

[43] Monteith GR, Davis FM, Roberts-Thomson SJ (2012). Calcium channels and pumps in cancer: changes and consequences. J Biol Chem.

[44] Muggen AF, Pillai SY, Kil LP, van Zelm MC, van Dongen JJ, Hendriks RW (2015). Basal Ca (2+) signalling is particularly increased in mutated chronic lymphocytic leukemia. Leukemia.

[45] Chen SS, Claus R, Lucas DM, Yu L, Qian J, Ruppert AS (2011). Silencing of the inhibitor of DNA binding protein 4 (ID4) contributes to the pathogenesis of mouse and human CLL. Blood.

[46] Grcević D, Marusić A, Grahovac B, Jaksić B, Kusec R (2003). Expression of bone morphogenetic proteins in acute promyelocytic leukemia before and after combined all trans-retinoic acid and cytotoxic treatment. Leuk Res.

[47] Dzietczenia J, Wróbel T, Jaźwiec B, Mazur G, Butrym A, Poręba R (2010). Expression of bone morphogenetic proteins (BMPs) receptors in patients with B-cell chronic lymphocytic leukemia (B-CLL). Int J Lab Hematol.

[48] Gilboa L, Nohe A, Geissendörfer T, Sebald W, Henis YI, Knaus P (2000). Bone morphogenetic protein receptor complexes on the surface of live cells: a new oligomerization mode for serine/threonine kinase receptors. Mol Biol Cell.

[49] Chen D, Ji X, Harris MA, Feng JQ, Karsenty G, Celeste AJ (1998). Differential roles for bone morphogenetic protein (BMP) receptor type IB and IA in differentiation and specification of mesenchymal precursor cells to osteoblast and adipocyte lineages. J Cell Biol.

[50] Hanoun M, Eisele L, Suzuki M, Greally J, Hu¨ttmann A, Aydin S (2012). Epigenetic silencing of the circadian clock gene *CRY1* is associated with an indolent clinical course in chronic lymphocytic leukemia. PLoS One.

[51] Yang MY, Chang JG, Lin PM, Tang KP, Chen YH, Lin HY (2006). Downregulation of circadian clock genes in chronic myeloid leukemia: alternative methylation pattern of hPER3. Cancer Sci.

[52] Liu Y, Miao Y, Wang J, Lin X, Wang L, Xu HT (2013). DEC1 is positively associated with the malignant phenotype of invasive breast cancers and negatively correlated with the expression of claudin-1. Int J Mol Med.

[53] Lee JC, Sharma M, Lee YH, Lee NH, Kim SY, Yun JS (2008). *PAX9* mediated cell survival in oral squamous carcinoma cell enhanced by c-myb. Cell Biochem Funct.

[54] Wielscher M, Vierlinger K, Kegler U, Ziesche R, Gsur A, Weinhäusel A (2015). Diagnostic performance of plasma DNA methylation profiles in lung cancer, pulmonary fibrosis and COPD. EBio Medicine.

[55] Gorbacheva VY, Kondratov RV, Zhang R, Cherukuri S, Gudkov AV, Takahashi JS (2005). Circadian sensitivity to the chemotherapeutic agent cyclophosphamide depends on the functional status of the CLOCK/BMAL1 transactivation complex. Proc Natl Acad Sci U S A.

[56] Kondratov RV, Kondratova AA, Lee C, GorbachevaVY CMV, Antoch MP (2006). Post-translational regulation of circadian transcriptional CLOCK(NPAS2)/BMAL1 complex by cryptochromes. Cell Cycle.

[57] Gauger MA, Sancar A (2005). Cryptochrome, circadian cycle, cell cycle checkpoints, and cancer. Cancer Res.

